# In Vitro Evaluation of Genetically Unmodified Ligand-Armed Allogeneic Natural Killer Cells to Treat EGFR-Positive Glioblastoma

**DOI:** 10.3390/cells14040254

**Published:** 2025-02-11

**Authors:** Hortense Courot, Emilie Rigal, Nawfel Adib, Marc Criton, Alan Cookson, Bénédicte Fauvel, Jessy Presumey

**Affiliations:** 1 CYTEA BIO, 34790 Montpellier, France; 2 MedXCell, 1820 Montreux, Switzerland

**Keywords:** natural killer cells, Fc-engineered antibodies, glioblastoma, EGFR

## Abstract

Glioblastomas (GBMs) are lethal brain tumors in which EGFR gene amplification or mutation is frequently detected and is associated with poor prognosis. The standard of care is maximal resection followed by chemotherapy and radiation. Over the last twenty years, marginal improvements in patient survival have been achieved mainly through surgical techniques and the more accurate use of radiation. In this study, umbilical cord blood-derived and expanded human allogeneic natural killer (eNK) cells were pre-complexed to an Fc-engineered anti-EGFR monoclonal antibody (Pin-EGFR) to create Pin-EGFR-armed eNK cells. Pin-EGFR-armed eNK cells showed in vitro persistence of mAb anchoring. This arming process mediated specific, rapid and potent NK cell-redirected cytotoxicity against GBM cell lines and patient-derived cells in models consistent with the pathophysiological conditions of GBM. These results demonstrate the potential of Pin-EGFR-armed eNK cells to be an effective therapy against GBM cell lines in vitro. This product represents a promising strategy to directly target residual tumor tissue remaining at and beyond the resection margins immediately following GBM surgery to improve patient care.

## 1. Introduction

Glioblastoma multiforme (GBM), or glioma grade 4, is the most common primary brain tumor in adults [1]. The first-line standard of care (SOC) consists of surgical debulking where surgically possible, followed by radiation and chemotherapy (temozolomide, TMZ) (NCCN, V1, 2023, [2]). Second-line treatments are more variable and generally seek to provide a better quality of life with milder adverse effects but do not significantly improve overall survival time. GBM remains an incurable disease with a median survival of 15 months from diagnosis with the current SOC, highlighting the urgent need to identify new therapeutic options with the aim to extend patient survival and reduce the adverse effects of standard treatments [3]. Most GBM surgeries have focused on the resection of contrast-enhanced tumors. Nevertheless, in recent years, with evidence that most recurrences originate from the peri-resection cavity due to the presence of infiltrative tumor cells, extending the resection beyond the borders of the contrast-enhanced tumor (supramarginal resection) has led to patient outcome improvements. Indeed, some evidence suggests a survival benefit with supramarginal resection without significant additional morbidity. These findings warrant further consideration surrounding this therapeutic niche, where the development of alternative therapeutic interventions will improve the removal of normal-appearing but malignant unexcised tissue [4,5,6].

In addition to the challenges of surgery, GBM is a complex and heterogeneous disease with great variability at the molecular and clinical levels, raising the need for tailored and targeted therapeutic approaches that can meet the specific features of GBM subtypes. Among the protein markers expressed preferentially on GBM cells that could be targeted by therapeutic modalities, several studies have focused on EGFR (epidermal growth factor receptor), either the wild type or its variants, mainly EGFRvIII (EGFR variant 3, in-frame deletion of exons 2 to 7). EGFR belongs to the ErbB family of receptor tyrosine kinases (RTKs) and exerts critical functions in epithelial cell physiology. It is frequently mutated and/or overexpressed in different types of human cancers and is the target of multiple cancer therapies currently adopted in clinical practice [7]. In GBM tumors, EGFR overexpression or EGFR mutations contribute to and alter the biology of the tumor and are highly prevalent, occurring in more than 50% of GBM cases [8,9,10]. Targeted therapies, including cell-based immunotherapies, against this receptor have consequently been tested in clinical settings for GBM. Nevertheless, most of them have shown limited activity and failed to demonstrate significant clinical benefit in the GBM patient population [11,12,13]. As an example, cetuximab, an approved anti-EGFR (targeting both wild-type EGFR and EGFRvIII [14]) monoclonal antibody (mAb), administered systemically, was well tolerated but had limited activity and failed to demonstrate benefit in the progressive high-grade glioma (HGG) patient population [11]. One major factor that could have contributed to the poor response of glioblastoma patients to cetuximab and other immunotherapies is ineffective blood–brain barrier (BBB) penetration associated with low intratumoral T-cell infiltration, with glioblastoma being considered an immunologically ‘cold’ tumor [15,16].

Natural killer (NK) cells are innate killer cells that can naturally eliminate virus-infected or transformed cells without prior sensitization. NK cells are also able to induce antibody-dependent cellular cytotoxicity (ADCC), mainly through the CD16a (Fc**γ**R3a) cell surface receptor that binds to the Fc portion of immunoglobulin G (IgG) antibodies. Their use in cell therapy presents several advantages, including multiple potential tissue sources and the absence of graft-versus-host (GVH) reaction. The potential of NK cells as effectors against brain tumors has been demonstrated in vitro [17,18,19,20] and in vivo [21,22,23]. Clinical trials using NK cells against brain tumors have already been conducted, demonstrating their safety, but their anti-tumor effects were limited [24,25]. To increase their effectiveness and to target specific tumor-associated antigens, NK cells may be modified with CAR (chimeric antigen receptor) to generate CAR-NK cells or combined with immunotherapy to take advantage of their ADCC function [26,27,28,29]. For the latter, the combination of therapeutic doses of cetuximab, known to selectively promote ADCC against EGFR-positive cells [30,31], with adoptive NK cells has been evaluated in various EGFR-expressing cancers. This combination was well tolerated, and early data showed better outcomes in patients (NCT02845856, NCT02845999, NCT03319459 and NCT04872634), and further studies should confirm these results (NCT05040568, NCT05069935 and NCT05395052).

We developed the Pin platform, introducing specific mutations in the Fc region of mAbs. These Fc-engineered mAbs exhibit the improved engagement of CD16, leading to the long-term noncovalent stabilization of the mAb onto the NK plasma membrane. These armed NK cells can be prepared upon request or cryopreserved to generate multiple off-the-shelf therapeutic products with dedicated targeting to a large spectrum of diseases. The platform has already been successfully applied to the rituximab backbone (Fc-engineered rituximab, Pin-CD20), with armed NK cells exhibiting significant and specific in vitro and in vivo cytotoxicity against CD20-expressing cancer cells [32].

Here, we applied the platform to a cetuximab backbone to generate the final product, Fc-engineered cetuximab (Pin-EGFR)-armed NK cells, and demonstrated its superior in vitro potency against EGFR-expressing cancer cells, focusing on glioblastoma cells. NK cells were expanded from umbilical cord blood units (eNK cells) and complexed with Pin-EGFR. Compared to unarmed eNK cells, this arming process led to the manufacturing of a stronger specific and cytotoxic product against both conventional GBM cell lines and patient-derived GBM cells. Moreover, cell arming standardized cytotoxic potential, demonstrating less variability between batches and also between patient samples. We also elucidated that the mechanism of action of Pin-EGFR-armed eNK cells harnesses the natural ADCC process. Furthermore, Pin-EGFR-armed eNK cells are resistant to one of the most immunosuppressive factors of the GBM tumor microenvironment (TGF-β) while remaining viable and fully effective in the presence of the standard GBM chemotherapeutic drug (temozolomide). This study presents Pin-EGFR-armed eNK cells as the pioneer of a new class of modular cell therapy combining the cytotoxic effects of genetically unmodified allogeneic NK cells and the absolute specificity of mAbs for their target in a pre-formed complex ready to be activated upon contact with target-expressing cells. This drug may be utilized to induce residual tumor shrinkage concomitantly or very rapidly after GBM surgical resection.

## 2. Materials and Methods

### 2.1. Pin-EGFR Manufacturing

**Figure 1:**
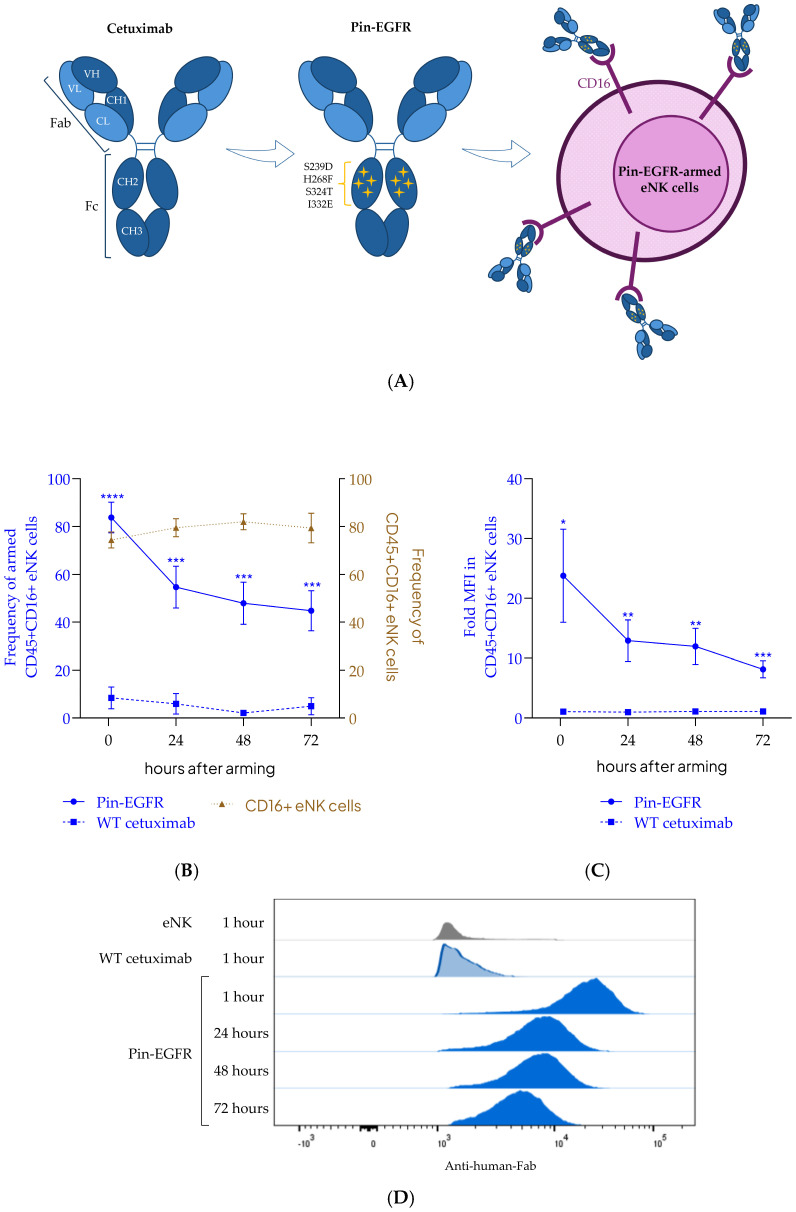
Long-term binding of Pin-EGFR on eNK cells. (**A**) Schematic illustration of the Pin-EGFR monoclonal antibody and the preparation of Pin-EGFR-armed eNK cells. The two light chains of the mAb are in light blue, each with one variable (VL) and one constant (CL) domain. The two heavy chains are in dark blue, each with one variable (VH) domain followed by a constant domain (CH1), a hinge region, and two more constant (CH2 and CH3 domains). Fab: fragment, antigen binding; Fc: fragment, crystallizable region. Pin-EGFR mutations in the CH2 domain of the Fc part are represented by yellow stars. (**B**–**D**) eNK cells were incubated with Fc-engineered (solid blue line, Pin-EGFR) or wild-type cetuximab (dotted blue line, WT cetuximab) at 10 μg/mL for 1 h at 37 °C. Cells were then washed twice and incubated at 37 °C for 24 h, 48 h or 72 h. At the indicated hours, flow cytometry was used to analyze CD16 expression frequency and mAb binding on eNK cells using an anti-human-Fab detection antibody. The frequency of viable CD45+/CD56+/CD16+ eNK cells (right y axis, brown) and CD45+/CD56+/CD16+ armed eNK cells (left y axis, blue) are shown in (**B**). The fold-change in the geometric mean of fluorescence intensity (gMFI) detected by anti-Fab in CD45+/CD56+/CD16+ eNK cells normalized to unarmed eNK cells is shown in (**C**). Data are representative of 13 individual donors for CD16 and Pin-EGFR (frequency and gMFI) and 6 individual donors for wild-type cetuximab. Welch’s unequal variances t-test was applied to analyze differences between Pin-EGFR-armed eNK and WT cetuximab-armed eNK groups; mean ± SEM is shown; **** *p* < 0.0001, *** *p* < 0.001, ** *p* value < 0.01, * *p* value < 0.05. A representative histogram plot for one eNK donor is shown in (**D**).

Pin-EGFR (Figure 1A), a novel human IgG1 with the Fab sequence of cetuximab and an Fc region containing 4 amino acid substitutions in the upper CH2 (S239D/H268F/S324T/I332E), was produced by RD-Biotech (Besançon, France) in CHO cells and purified using protein A. All batches contained < 1 EU/mg of endotoxins.

### 2.2. Cells and Culture Conditions

Patient-derived cells were sourced and accessed for commercial use under a license agreement from QIMR Berghofer Medical Research Institute [33]. U-87 MG and U-251 MG cells were obtained from Sigma (Catalog #89081401-1VL and #09063001-1VL, Saint Louis, MO, USA) and cultured in DMEM (Gibco™ 31885023, Billings, MT, USA) containing 10% FBS (Gibco™ 10438026) and 1% penicillin–streptomycin (Gibco™ 15140122). Patient-derived cells were cultured in StemPro^TM^ NSC SFM (Gibco™ A1050901) with all supplements according to the manufacturer’s instructions and supplemented with 1% penicillin–streptomycin (Gibco™ 15140122), except BAH1, for which EGF was not added to the culture medium. The cells were cultured and kept at a temperature of 37 °C with 5% CO_2_. Peripheral blood mononuclear cells (PBMCs) were isolated using a conventional density gradient method (Lymphoprep™ (Oslo, Norway), and SepMate^TM^ (Temecula, CA, USA), Stemcell) from fresh blood samples from healthy donors for research purposes supplied by the French Blood Transfusion Center (Cell and Tissue Engineering Activity, EFS Besançon, Besançon, France). Written informed consent was obtained from all donors.

### 2.3. Generation of eNK Cells

The eNK expansion protocol was derived from a previously described protocol [32]. Fresh umbilical cord blood (UCB) units for research purposes were obtained from the French Blood Transfusion Center Cord Blood Bank (Cell and Tissue Engineering Activity, EFS Besançon, Besançon, France). Written informed consent was obtained from all donors. Briefly, negative selection (EasySep™ Human CD3 Positive Selection Kit II, Stemcell, Vancouver, BC, Canada) was performed on donor umbilical cord blood mononuclear cells to purify CD3 negative cells, according to the manufacturer’s instructions. CD3-negative cells were seeded into a gas-permeable cell culture device (G-Rex 6M, Wilson–Wolf Manufacturing, Saint Paul, MN, USA) at 0.5 × 10^6^ cells/cm^2^ in eNK medium (NK MACS^®^ medium, Miltenyi, Bergisch Gladbach, Germany) supplemented with 5% human serum (specific batch manufactured from EFS Besançon, Besançon, France), 100 IU/mL rhIL-2 (Peprotech, GMP200-02, Cranbury, NJ, USA) and 50 IU rhIL-15 (Peprotech, GMP200-15). On day 7, eNK cells were transferred to 100 M G-Rex, and freshly prepared eNK medium was added to reach a volume of 1 L. Then, 70 Gy-irradiated lymphoblastoid BK-EBV feeder cells (the cells were supplied by the Cellular and Gene Therapy Unit of the Nantes University Hospital, France) at appropriate ratios were added on day 0 and day 7. On day 11, cell purity, viability and phenotype were checked by flow cytometry before cryopreservation in CryoStor^®^ CS10 (Stemcell).

### 2.4. Arming of eNK Cells and Kinetics of Binding

The day before arming, eNK cells were thawed and cultured in RPMI 1640 Glutamax (Gibco^TM^ 61870044) containing 10% FBS (Gibco™ 10438026). eNK cells were counted using a Muse Cell Analyzer (CYTEK) and then incubated at 10 × 10^6^ cells/mL in RPMI 1640 Glutamax with 10 µg/mL of Pin-EGFR mAbs for 1 hour at 37 °C and 5% CO_2_. The concentration of Pin-EGFR mAbs was selected according to a previously published work [32]. eNK cells were next washed twice with RPMI 1640 Glutamax to remove the excess of unbound Pin-EGFR mAbs. Pin-EGFR-armed eNK cells (Figure 1A) were then cultured in RPMI 1640 Glutamax (Gibco^TM^ 61870044) containing 10% FBS (Gibco™ 10438026). The binding of Pin-EGFR to eNK cells was analyzed by flow cytometry immediately, 24 h, 48 h and 72 h after arming.

### 2.5. Flow Cytometry Analysis and Antigen Quantification

Next, 2 × 10^5^ cells were stained in PBS containing 2% FBS and 1 mM EDTA using the following FACS antibodies: CD56-BV451 (362552, Biolegend, San Diego, CA, USA, 1/100); CD45-BV510 (304036, Biolegend, 1/100); CD16-PerCP-Cy5.5 (302028, Biolegend, 1/100); NKp30-PE-Cy7 (325213, Biolegend, 1/100); NKG2A-AF488 (375123, Biolegend, 1/100); NKG2C-PE (375003, Biolegend, 1/100); NKG2D-APC (320808, Biolegend, 1/100); CD2-PB clone RPA-2.10 (300235, Biolegend, 1/100); NKp46-FITC (331922, Biolegend, 1/100); NKp44-PE (325127, Biolegend, 1/100); and CXCR4-PerCP-Cy5.5 (306515, Biolegend, 1/100). Cells were incubated for 20 min at 4 °C in the dark and washed using PBS containing 2% FBS and 1 mM EDTA. The binding of Pin-EGFR to eNK cells was analyzed in 2 × 10^5^ cells stained in PBS containing 2% FBS and 1 mM EDTA with the following FACS antibodies: CD56-PE-Cy7 (392411, Biolegend, 1/100); CD45-VioGreen (360712, Biolegend, 1/100) or CD45-BV510 (304036, Biolegend, 1/100); CD16-PerCP-Cy5.5 (302028, Biolegend, 1/100); and anti-human Fab-AF647 (CSA3835, Cohesion Biosciences, London, UK, 1/500). The analysis of EGFR expression frequency in target cells was performed on 2 × 10^5^ .cells from cell lines or patient-derived cells stained in PBS containing 2% FBS and 1 mM EDTA with anti-EGFR-PE antibodies (555997, BD Biosciences, Franklin Lakes, NJ, USA). Cells were incubated for 20 min at 4 °C in the dark and washed using PBS containing 2% FBS and 1 mM EDTA. For all analyses, eFluor 780 dye (65-0865, Invitrogen, Waltham, MA, USA, 1/10,000) was used to discriminate dead from live cells. Cells were then resuspended in the same buffer and analyzed on a Northern Lights flow cytometer (Cytek). The EGFR absolute number of molecules was quantified using a BD Quantibrite™ PE Phycoerythrin Fluorescence Quantitation Kit (BD Biosciences) according to the manufacturer’s instructions. Briefly, 2 × 10^5^ cells were stained with 5 µL of PE-conjugated anti-EGFR FACS antibody (555997, BD Biosciences). After 20 min incubation at 4 °C in the dark, cells were washed, resuspended in PBS containing 2% FBS and 1 mM EDTA and analyzed on a Northern Lights flow cytometer (Cytek). The antigen absolute number was calculated according to the manufacturer’s instructions.

### 2.6. Cytotoxicity Assays

For adherent cell lines (U-87 MG and U-251 MG), target cell viability was measured using classical MTT (M2128, Sigma, Saint Louis, MO, USA) or CCK-8 (MedChemExpress, Monmouth Junction, NJ, USA, HY-K0301) assays. For adherent patient-derived cells (FPW1, MN1 and MMK1), target cell viability was measured using a classical CCK-8 assay. The day before, target cells were plated in 96-well plates at 1 × 10^4^ cells per well. The day of the experiment, eNK cells were armed with Pin-EGFR mAbs and added to wells at a corresponding effector-to-target (E:T) ratio (a 3:1 E:T means 3 eNK cells for 1 target cell). For the classical ADCC assay, mAbs were also co-incubated with U-87 MG target cells for 10 min before adding unarmed eNK cells at a 3:1 E:T ratio. After 24 h of co-culture at 37 °C and 5% CO_2_, wells were washed with PBS and MTT or CCK-8 reagent was added according to the manufacturer’s instructions. Light absorbance was measured at 560–655 nm for the MTT method and 450 nm for the CCK-8 method using a SpectraMax iD3 spectrophotometer (Molecular Devices). The measured absorbances were normalized to untreated target cell wells (100% viability) and Triton X100-treated wells (0% viability). In the experiment using human PBMCs from healthy donors, unarmed eNK cells or Pin-EGFR-armed eNK cells were stained with CFSE CellTrace dye (C34554, Invitrogen, 1µM) and co-incubated with PBMC from healthy donors at a eNK:PBMC ratio of 3:1 for 24 h. PBMCs were then gathered and stained using anti-CD3-APC/Fire 810 (344857, Biolegend, 1/100), anti-CD19-PE (561741, BD Pharmingen™, 1/100), anti-CD56-PE-Cy7 (392411, Biolegend, 1/100) and viability dye eFluor 780 dye (65-0865, Invitrogen, 1/10,000) and analyzed on a Northern Lights (Cytek) flow cytometer to discriminate T cells, B cells, NK cells and NKT cells. For GBM patient-derived cells in suspension (RKI1, PB1, RN1 and BAH1), target cell viability was analyzed by flow cytometry. The day before, target cells were plated in 96-well plates at 1.5 × 10^4^ cells per well. eNK cells were labeled for 20 min at 37 °C with CFSE CellTrace dye (C34554, Invitrogen, 1 µM) before washing and plating. CFSE-labeled eNK cells were armed with Pin-EGFR mAbs and added to the wells at a corresponding E:T ratio. After 24 h of co-culture at 37 °C and 5% CO_2_, the volume of each well was transferred to a new 96-well plate, and cells were analyzed by flow cytometry analysis using absolute cell count by adding beads (Precision count beads, 424902, Biolegend, San Diego, USA). The target cell count for each well was normalized to the mean cell count of the untreated target cell wells (100% viability).

### 2.7. Live Kinetic Analysis of Tumor Cell Killing

U-87 MG cells were labeled with Cytolight Green solution (Sartorius (Göttingen, Germany) 4705, 110 nM, 20 min, at 37 °C). For the kinetic analysis of tumor cell killing, labeled U-87 MG cells were plated at a concentration of 1 × 10^4^ cells per well in 96-well flat bottom plates. The following day, eNK cells were armed by mixing with either PBS (unarmed) or 10 µg/mL of Pin-EGFR mAbs and added at an E:T ratio of 3:1. All co-cultures were performed in RPMI medium. The number of viable target cells was monitored by hourly fluorescence imaging over 24 h using an IncuCyte Live Cell Analysis System (Sartorius, Göttingen, Germany). Live cell numbers were quantified using IncuCyte software (version 2023A, Sartorius) and normalized to the number of live cells at t0 in the target cell-only control group (100% live cells).

### 2.8. Cytokine Secretion

IFN-γ and TNF-α secretion was analyzed in the presence or absence of target cells (U-87 MG). Target cells were plated (1 × 10^5^ cells per well), and the following day, eNK cells were armed with Pin-EGFR mAbs and added at an E:T ratio of 3:1. After 24 h, the supernatant of each well was collected and analyzed by flow cytometry using Lumit IFN-γ and Lumit TNF-α Immunoassay kits (W6040/W6050, Promega, Madison, WI, USA) according to the manufacturer’s instructions.

### 2.9. TGF-β and TMZ Effects on eNK Cells

Carrier-free recombinant human TGF-β (781802, Biolegend) or temozolomide (TMZ, HY1754, MedChemExpress, Monmouth Junction, NJ, USA) was used at increasing concentrations. eNK-cell viability was assessed after 24 h of exposure to TGF-β (0.1, 1 and 10 ng/mL) or 24 h and 72 h exposure to TMZ (0.1, 1, 10 and 100 µM) by flow cytometry (eNK stained with eFluor 780 (65-0865, Invitrogen, Waltham, MA, USA)). The cytotoxic potential of eNK cells in the presence of TGF-β or TMZ was assessed for 24 h using same increasing concentrations (0.1, 1 and 10 ng/mL for of TGF-β and 0.1, 1, 10 and 100 µM for TMZ) when co-cultured with target cells (U-87 MG) at an E:T ratio of 3:1 by CCK-8 assay.

### 2.10. Statistical Analysis

Quantitative data are represented as the mean and standard error of the mean (SEM). To determine statistical significance (* *p* value < 0.05, ** *p* value < 0.01, *** *p* value < 0.001, **** *p* value < 0.0001), Welch’s unequal variances t-test, one-way ANOVA with the Kruskal–Wallis test, two-way ANOVA with Tukey’s test or the Mann–Whitney test was applied. For distribution analysis, a Wilcoxon–Mann–Whitney test was applied. All analyses were performed with Prism 10 software (GraphPad Software, version 10.4.1).

## 3. Results

### 3.1. Evaluation of Long-Term Arming of Pin-EGFR on eNK-Cell Surface

The binding of Pin-EGFR (Fc-engineered cetuximab) on eNK cells from multiple separate donors was evaluated over time by flow cytometry to assess the long-term arming at the cell surface. Briefly, the persistence of Pin-EGFR on eNK cells was compared to the persistence of wild-type cetuximab (WT cetuximab). As shown in Figure 1B, 1 h after arming, more than 80% of eNK cells were detectably armed with Pin-EGFR. Whereas CD16 expression remained stable, the arming (frequency and geometric mean of fluorescence intensity (gMFI) of Pin-EGFR eNK (Figure 1B,C)) decreased for 24 h (30% of eNK cells had undetectable arming after 24 h) and then continued to decrease but more slowly over time (loss of approximately 10% of detectable arming on eNK cells in the two following days), with more than 40% eNK cells remaining detectably armed after 3 days in culture. WT cetuximab, after only 1 h of incubation, was not able to stay attached to the eNK-cell surface.

We thus demonstrated that Pin-EGFR mAbs exhibit persistent and stable binding to eNK cells.

### 3.2. Cytotoxicity Exhibited by Pin-EGFR-Armed eNK Cells Against Glioblastoma Cell Lines In Vitro

The in vitro potency of Pin-EGFR-armed eNK cells was investigated on cells expressing the recognized specific antigen (EGFR). The ADCC efficiency of Pin-EGFR was first assessed using armed eNK cells on conventional GBM cancer cell lines (U-87 MG and U-251 MG).

**Figure 2:**
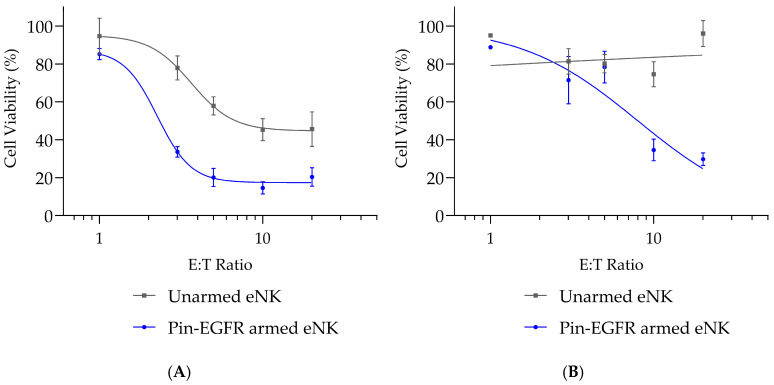

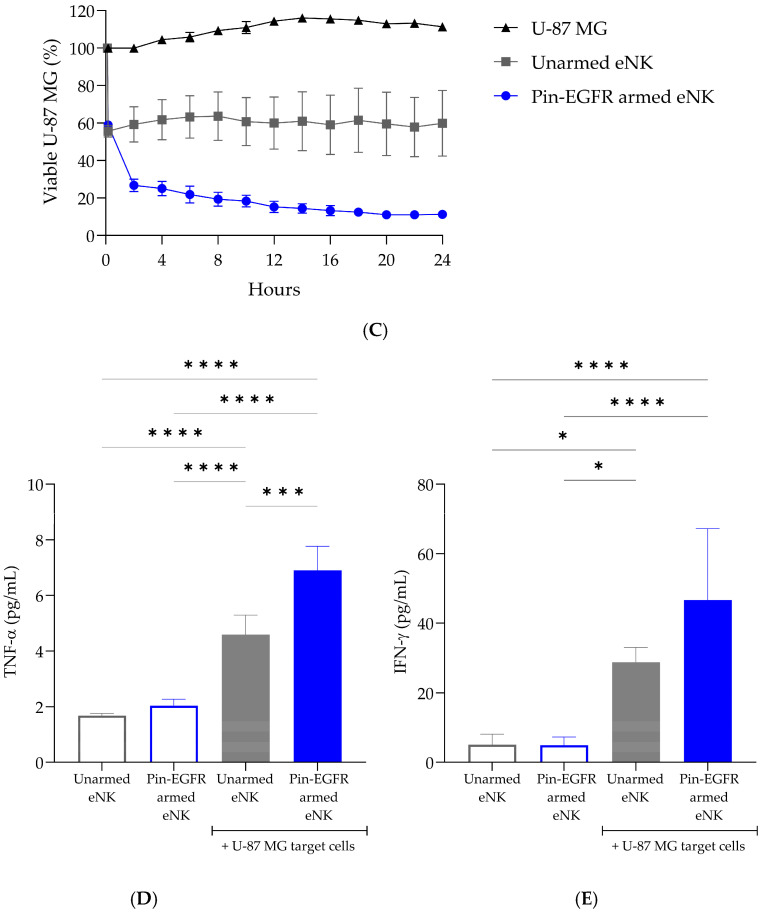
Ratio- and time-dependent specific pharmacological effects of Pin-EGFR-armed eNK cells on GBM cell lines. The day before, GBM cancer cells (U-87 MG or U-251 MG) were plated. eNK cells (*n* = 7 donors) were armed with 10 µg/mL Pin-EGFR and co-cultured with the adherent glioblastoma cells. The ratio-dependent pharmacological effect of Pin-EGFR-armed eNK cells on U-87 MG (**A**) or U-251 MG (**B**) cells was evaluated at 24 h using an increasing effector-to-target (E:T) ratio. Unarmed eNK cells were used as controls for natural cytotoxicity. Target cell viability was measured using a classical MTT assay. (**C**) The time-dependent pharmacological effect of Pin-EGFR-armed eNK cells was evaluated on U-87 MG cells at an E:T ratio of 3:1 using an IncuCyte Live Cell Analysis System (Sartorius). The day before, cancer cells were labeled and plated. eNK cells from donors (*n* = 1) were armed for 1 h at 37 °C with 10 µg/mL Pin-EGFR before co-culture with target cells. eNK cells and target cells were co-incubated for 24 h at an E:T ratio of 3:1. Unarmed eNK cells were used as controls for natural cytotoxicity. Target cell viability was measured by counting the number of U-87 MG cells per well normalized to the number of U-87 MG-untreated cells at time 0. Data are represented as mean ± SEM calculated in triplicate. Next, secretion of TNF-α (**D**) or IFN-γ (**E**) from unarmed eNK or Pin-EGFR-armed eNK cells was assessed in the presence or absence of U-87 MG cells at an E:T ratio of 3:1 (24 h of co-culture). *n* = 5 eNK donors; two-way ANOVA with Tukey’s test; mean ± SEM is shown. **** *p* < 0.0001, *** *p* < 0.001, * *p* value < 0.05.

The specific killing induced by armed eNK cells was compared to the natural cytotoxicity of unarmed eNK cells with an increasing effector-to-target (E:T) ratio (Figure 2). A ratio-dependent increase in cell death was observed, with unarmed eNK cells mainly for the U-87 MG cell line, demonstrating that this cell line is more sensitive compared to U-251 MG to natural NK cell-induced in vitro cytotoxicity (Figure 2A,B).

With a 5:1 E:T ratio, the maximal pharmacological effect of Pin-EGFR-armed eNK cells seemed to have been reached on U-87 MG cells, while an E:T ratio of 10:1 was required to maximize U-251 MG cell killing. Virtually all eNK natural cytotoxicity and ADCC effects induced by Pin-EGFR-armed eNK cells were observed within the first hours of co-culture with U-87 MG target cells (Figure 2C).

Moreover, in the absence of U-87 MG target cells, Pin-EGFR-armed eNK cells did not show elevated cytokine secretion compared with unarmed eNK cells (Figure 2D,E), demonstrating that the presence of the Pin-mAb attached to the CD16 receptor does not affect the basal steady state of eNK cells. However, when co-cultured with U-87 MG target cells, Pin-EGFR-armed eNK cells secreted significantly more pro-inflammatory cytokines (tumor necrosis factor (TNF)-α (Figure 2D) and interferon (IFN)-γ (Figure 2E)). Taken together, these results demonstrate that Pin-EGFR arming allows eNK cells to engage with their intended target protein on target cells to induce very rapid and efficient cytotoxicity with a stronger cytotoxic effect than that of unarmed eNK cells.

### 3.3. Specific ADCC Induced by Pin-EGFR-Armed eNK Cells

**Figure 3:**
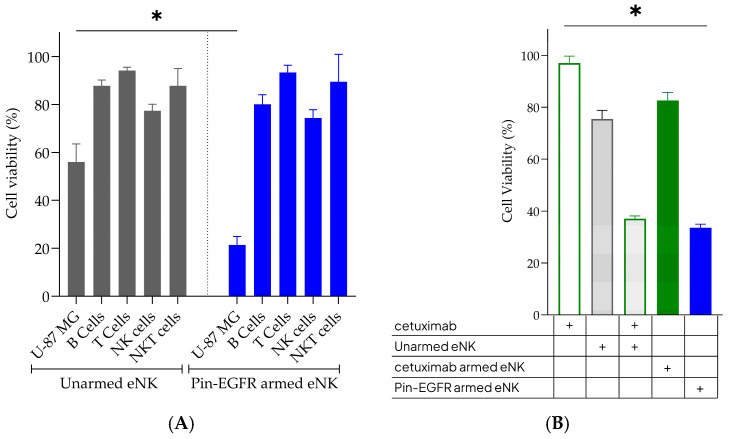
Specificity of Pin-EGFR-armed eNK cells to induce ADCC. (**A**) Target specificity of Pin-EGFR-armed eNK cells. The cytotoxicity of Pin-EGFR-armed eNK cells (*n* = 2 eNK donors, blue bars) was analyzed on EGFR-positive target cells U-87 MG and human PBMC cell types (B cells, T cells, NK cells, NKT cells) from healthy donors (*n* = 2 PBMC donors) during 24 h at an E:T ratio of 3:1. Unarmed eNK cells (grey bars) were used as controls for natural cytotoxicity. U-87 MG cell viability was measured using a classical CCK-8 assay. PBMC populations were assessed by flow cytometry: B cells (CD19+), T cells (CD56-/CD3+), NK cells (CD3-/CD56+) and NKT cells (CD3+/CD56+). Mann–Whitney test; mean ± SEM is shown. * *p* value < 0.05. (**B**) Comparison of classical ADCC with cytotoxicity induced by Pin-EGFR-armed eNK cells. The day before, U-87 MG cells were plated. eNK cells (*n* = 1 donor) were armed with 10 µg/mL Pin-EGFR (blue bar) or WT cetuximab (green bar) and co-cultured with the adherent glioblastoma cells at an E:T ratio of 3:1. Unarmed eNK cells (grey bar with grey border) were used as controls for natural cytotoxicity. WT cetuximab was opsonized for 10 min on U-87 MG before unarmed eNK cells (grey bar with green border) were added at the same E:T ratio of 3:1. U-87 MG cell viability was evaluated at 24 h by a classical CCK-8 assay. One-way ANOVA with Kruskall–Wallis test; mean ± SEM is shown. * *p* value < 0.05.

To better characterize the mechanism of action of Pin-EGFR-armed eNK cells, we first aimed at confirming that Pin-EGFR-armed eNK cells induce the specific killing of EGFR-expressing cells. U-87 MG target cells (expressing EGFR) and human PBMCs from healthy donors (not expressing EGFR) were treated with Pin-EGFR-armed eNK cells. Whereas U-87 MG cells were effectively targeted, none of the immune cell populations were altered by Pin-EGFR-armed eNK cells as compared to unarmed eNK cells (Figure 3A). This demonstration is consistent with the induction of an ADCC-like effect by Pin-EGFR-armed cells, redirecting eNK cytotoxicity to specifically target the mAb antigen. Consequently, in the case of armed cells, the antibody already attached to eNK cells (and not opsonized first on target cells before engaging the NK cells) seems capable of inducing the CD16-specific signaling pathway, enabling the well-known cascade involved in ADCC process.

We then compared the potency of this ADCC-like process (induced by armed eNK cells) with classical ADCC (induced by mAb opsonization on target cells). At a 10 µg/mL concentration, conventionally used to perform ADCC assays, WT cetuximab alone had no effect on U-87 MG cell viability (Figure 3B). On the other hand, in the presence of eNK cells, WT cetuximab induced much more pronounced cytotoxicity than the natural cytotoxicity of the cells, demonstrating an ADCC effect. This effect was no longer visible when the arming process was performed with WT cetuximab, in agreement with the inability of WT cetuximab to arm eNK cells (Figure 1A). Pin-EGFR-armed eNK cells induced similar ADCC-like potency to the ADCC effect of WT cetuximab. As the final step of the arming process involves two washes to remove excess antibody, we hypothesized that the actual amount of pin-EGFR present in the armed cells was probably less than the concentration of 10 µg/mL. A customized ELISA was set up and validated, and the actual concentration of Pin-EGFR mAb anchored onto the eNK-cell surface at the E:T ratio of 3:1 used for in vitro cytotoxicity assays was defined as 1.5 ng/mL. Although this will have to be validated in the future by evaluating the ADCC of cetuximab at lower concentrations, this result allows us to hypothesize that the ADCC-like properties of armed eNK cells require profoundly less mAb than classical ADCC induced by WT mAbs.

Taken together, these results elucidate the potential mechanism of action of Pin-EGFR-armed eNK cells. The strong binding of Pin-EGFR to CD16 allows for the powerful and specific activation of an ADCC process. This ADCC-like effect requires very low levels of pre-complexed mAbs compared to the required mAb quantities if mAbs and eNK cells are used separately (as is most often the case in therapeutic trials). In addition, the very low level of pre-complexed mAb ensures that the release and persistence of the antibody in patients will not give rise to unmanageable side effects.

### 3.4. Cytotoxicity Exhibited by Pin-EGFR-Armed eNK Cells Against Primary Glioblastoma Cells In Vitro

**Figure 4:**
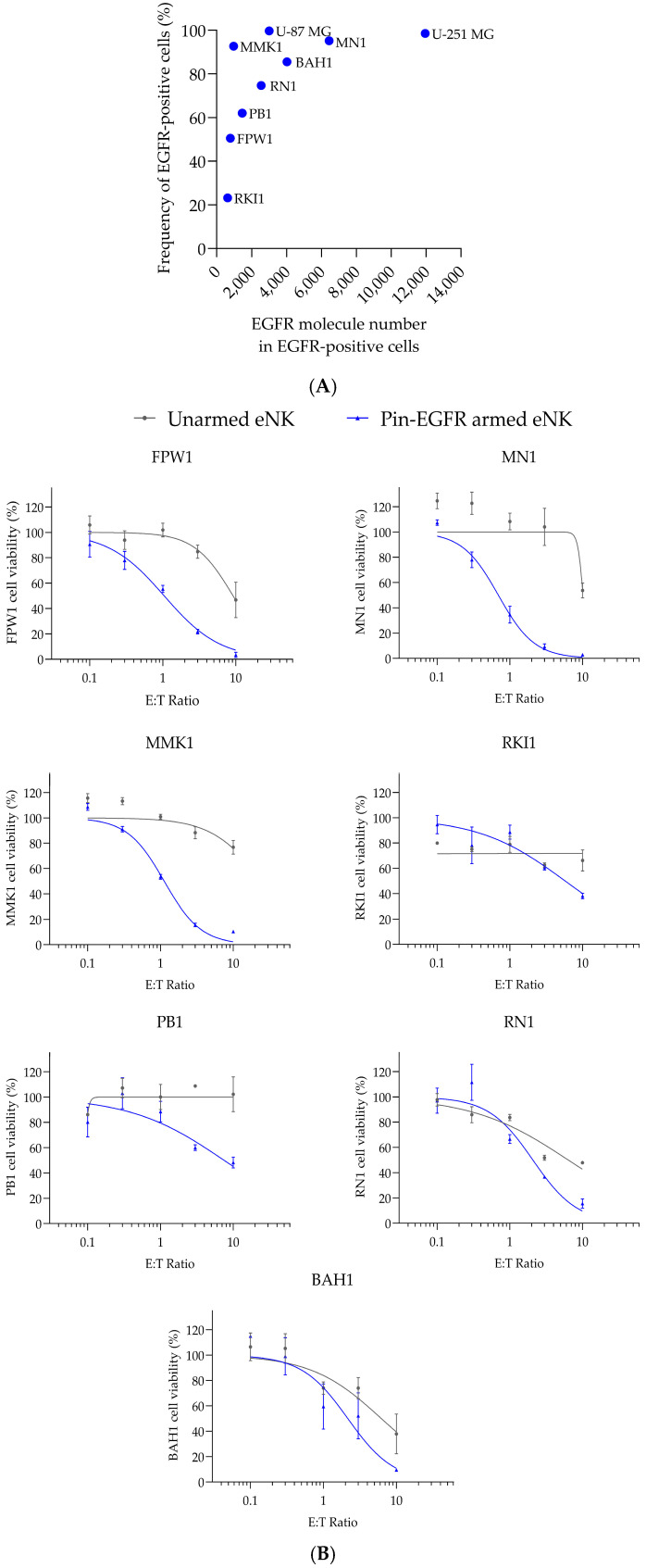

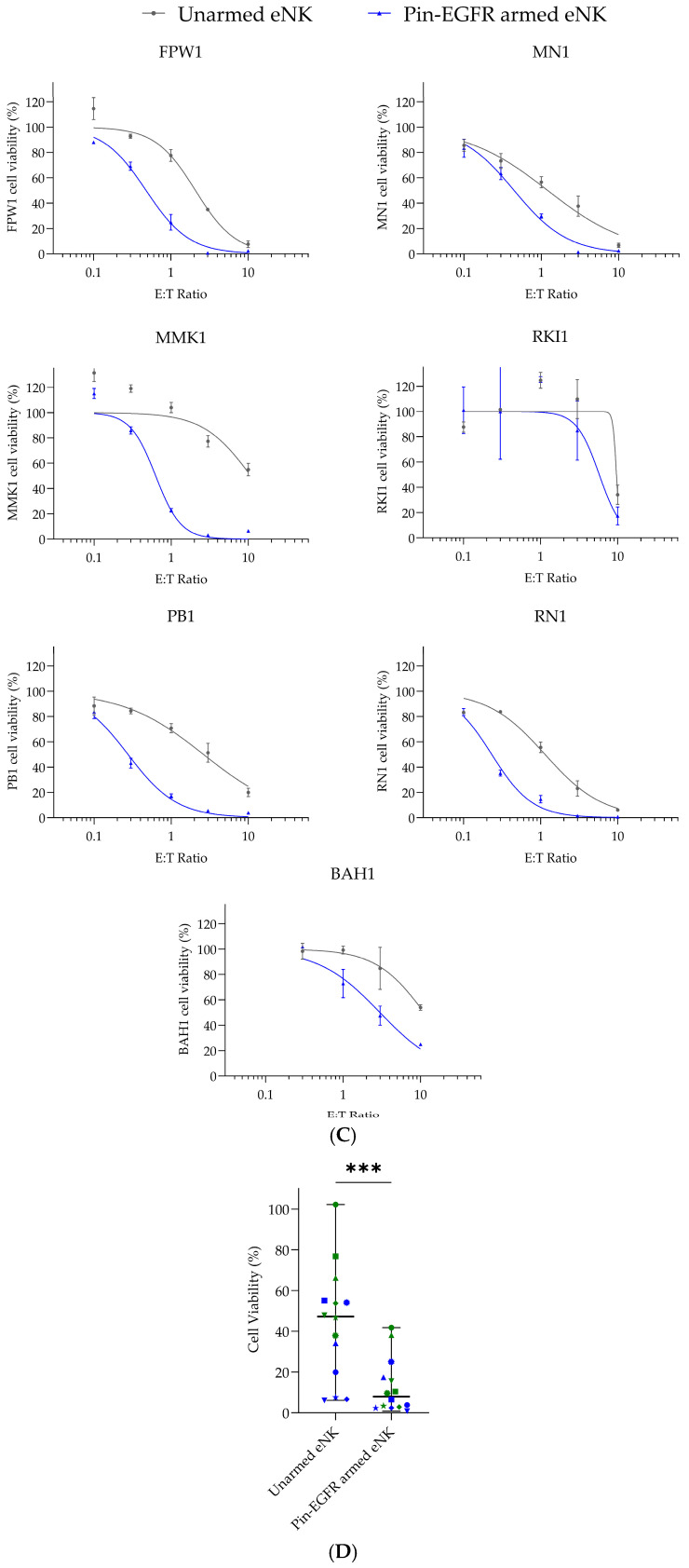
Ex vivo cytotoxicity of Pin-EGFR-armed eNK cells against patient-derived GBM cells. (**A**) EGFR expression levels were analyzed by flow cytometry and the number of EGFR molecules per cell estimated using Quantibrite^TM^ beads. (**B**,**C**) GBM cells were plated the day before. eNK cells from donor 1 (**B**) or donor 2 (**C**) were armed with 10 µg/mL Pin-EGFR and co-cultured with GBM cells. The dose-dependent pharmacological effect of Pin-EGFR-armed eNK cells was evaluated at 24 h using an increasing E:T ratio. Unarmed eNK cells were used as controls for natural cytotoxicity. Target cell viability was measured using a classical CCK-8 assay for patient-derived adherent cells (FPW1, MN1 and MMK1) or flow cytometry for GBM cells in suspension (RKI1, PB1, RN1 and BAH1). Data are represented for each eNK-cell donor with mean ± SEM calculated in duplicate. (**D**) The cell viability for every patient-derived cell (FPW1: star, MN1: diamond, MMK1: square, RKI1: triangle with base on the bottom, PB1: circle, RN1: triangle with base on the top and BAH1: sun) is depicted at an E:T ratio of 10:1 for donor 1 (symbols in green) and donor 2 (symbols in blue). Mann–Whitney test; median ± range is shown. *** *p* value < 0.001.

Seven patient-derived, genetically diverse, tumorigenic, low-passage, serum-free cell lines with a spectrum of cell morphologies were selected [33]. In addition to the known genotypic and phenotypic heterogeneity, this panel of patient-derived cells showed heterogeneity for EGFR expression (Figure 4A). The range of EGFR expression varied from 23% to 95% of positive cells, and the number of EGFR molecules on the surface of each cell, on average, varied from 600 (RKI1) to 6500 (MN1). Therefore, this cell panel mimicked the heterogeneity observed in tissue from GBM patients and was more accurate and reliable physiologically than classical GBM cell lines, which demonstrated very high EGFR surface expression (Figure 4A). The cytotoxicity of unarmed eNK cells and Pin-EGFR-armed eNK cells was evaluated after 24 h of co-incubation with an increasing E:T ratio on this cell panel. Pin-EGFR-armed eNK cells induced a strong increase in cytotoxicity compared to that of unarmed eNK cells on all primary GBM patient-derived cell lines tested with eNK cells manufactured from two individual donors (donor 1 data in Figure 4B, donor 2 data in Figure 4C), except for RKI1 cells. It should be noted that RKI1 has the lowest expression of EGFR. It is also important to highlight that a copy number gain of wild-type EGFR has been detected in RN1 and that BAH1 harbors an amplification of the oncogenic vIII variant of EGFR (EGFRvIII) [33]. Moreover, the natural cytotoxicity of eNK cells (unarmed eNK) is strongly donor dependent and patient-derived cell dependent (Figure 4D). This variability at a fixed E:T ratio ranges from no cytotoxicity (donor 1 on PB1) to more than 90% cytotoxicity (donor 1 on FPW1, MN1 and RN1). It should be noted that this cytotoxicity distribution is strongly and statistically reduced when eNK cells are armed with Pin-EGFR, ranging from 60% (donor 2 on RKI1 and PB1) to almost 100% (donor 1 on FPW1, MN1, PB1 and RN1 and donor 2 on FPW1, MN1 and BAH1).

The observed specific activity of Pin-EGFR-armed eNK cells on multiple patient-derived GBM cells suggests that this product may be a new therapeutic option for patients with glioblastoma expressing EGFR or the EGFRvIII variant or diagnosed with an amplification of wild-type EGFR. Arming eNK cells with Fc-engineered monoclonal antibody leads to a reduction in inter-donor and inter-patient variability in therapeutic responses.

### 3.5. Phenotype and Functionality of Pin-EGFR-Armed eNK Cells in Presence of TGF-β

**Figure 5:**
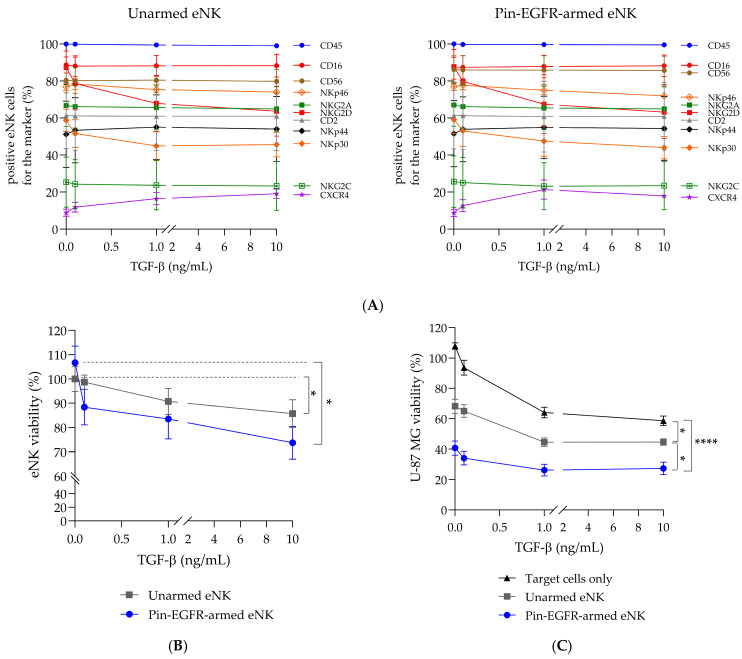
Evaluation of the dose-dependent effect of TGF-β on Pin-EGFR-armed eNK-cell phenotype, viability and functionality. The same eNK cells (*n* = 3 donors) were used for A, B and C. (**A**) eNK cells from donors (*n* = 3) were mixed at 37 °C with PBS (unarmed eNK cells) or 10 µg/mL Pin-EGFR and co-cultured without or with increasing concentrations of TGF-β (0.1, 1 and 10 ng/mL) for 24 h. Marker expression (**A**) and viability (**B**) were analyzed by flow cytometry. (**C**) The day before, GBM cancer cells (U-87 MG) were plated. eNK cells from donors (*n* = 3) were armed for 1 h at 37 °C with 10 µg/mL Pin-EGFR before co-culture with target cells. eNK cells and target cells were co-cultured for 24 h at an E:T ratio of 3:1 without or with increasing concentrations of TGF-β (0.1, 1 and 10 ng/mL). Target cell viability was measured using a classical CCK-8 assay. Two-way ANOVA with Tukey’s test; mean ± SEM is shown. **** *p* < 0.0001; * *p* value < 0.05.

Transforming growth tactor (TGF)-β has been shown to be a potent suppressor of NK-cell anti-tumor activity, including NK-cell ADCC, by inhibiting cytotoxicity, cytokine secretion, and NK-cell proliferation [34,35,36]. In GBM, tumor-infiltrating NK cells acquired an altered phenotype associated with impaired lytic function relative to matched peripheral blood NK cells from patients with GBM or healthy donors. This immune evasion tactic was attributed to the cell-to-cell contact between glioblastoma stem cells (GSCs) and NK cells via αv integrin-mediated TGF-β activation [37]. The impact of TGF-β on the phenotype, viability and functionality of unarmed eNK and Pin-EGFR-armed eNK cells was investigated in vitro (Figure 5). First, unarmed eNK or Pin-EGFR-armed eNK cells were co-cultured for 24 h with increasing concentrations of TGF-β, and phenotypic markers expression was analyzed by flow cytometry. No statistical difference was observed comparing either unarmed and armed eNK cells or both in the presence or absence of TGF-β (Figure 5A). Importantly, CD16 expression was not altered. Nevertheless, a small dose-dependent decrease in the frequency of cells expressing the activating receptors NKG2D and NKp30 associated with a small dose-dependent increase in the chemokine receptor CXCR4 were observed. This is in agreement with the literature [21,38,39,40]. Likewise, as previously described in the literature, TGF-β slightly affected eNK viability, whether armed or unarmed, with significant effects at the maximum concentration of 10 ng/mL compared with the viability observed without TGF-β (Figure 5B). Regarding functionality, the natural cytotoxicity of unarmed eNK cells and the ADCC properties of Pin-EGFR-armed eNK cells without TGF-β were in the range of historical data at an E:T ratio of 3:1 (see Figure 2A). It is noteworthy that, as previously described in the literature, U-87 MG cells seemed sensitive to increasing TGF-β concentrations (Figure 5C) [41]. Increasing concentrations of TGF-β slightly affected unarmed eNK cells and to a lesser extent armed eNK cells (respectively 23% and 13% less cytotoxicity observed in the presence of 10 ng/mL TGF-β compared to no TGF-β). These findings may be the result of either the direct inhibition of the cytotoxic potential of eNK cells, the consequence of the impact of TGF-β on their viability or both. Nevertheless, Pin-EGFR-armed eNK cells were still able to induce ADCC even at the maximum tested TGF-β concentration. To conclude, the stability of CD16 expression appears to guarantee the efficient ADCC function of Pin-EGFR-armed eNK cells in the presence of TGF-β, supporting the use of this product even in strongly immunosuppressive tumor microenvironments.

### 3.6. Behavior of Pin-EGFR-Armed eNK Cells in Presence of Temozolomide

**Figure 6:**
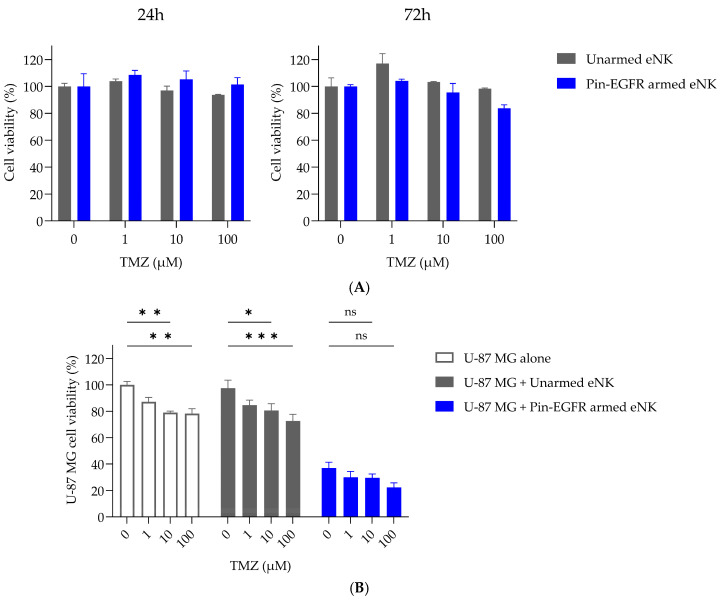
Time- and dose-dependent effects of TMZ on the viability and functionality of eNK cells. (**A**) eNK cells (*n* = 1 donor) were armed by mixing 10 µg/mL Pin-EGFR at 37 °C for 1 h and co-cultured without or with increasing concentrations of TMZ (1, 10 and 100 µM) for 24 h or 72 h. Cell viability was analyzed by flow cytometry. (**B**) The day before, GBM cancer cells (U-87 MG) were plated. eNK cells from donors (*n* = 2) were armed for 1 h at 37 °C with 10 µg/mL Pin-EGFR before co-culture with target cells. eNK cells and target cells were co-cultured at an E:T ratio of 3:1 without or with increasing concentrations of TMZ (1, 10 and 100 µM) for 24 h. U-87 MG cell viability was measured using a classical CCK-8 assay. Two-way ANOVA with Tukey’s test; mean ± SEM is shown. *** *p* < 0.001; ** *p* < 0.01; * *p* value < 0.05.

Alkylating agents such as temozolomide (TMZ), the primary chemotherapeutic agent for glioblastoma, methylates guanine residues, thereby inducing cascading cycles leading to cytotoxicity. However, the drug’s concomitant lymphodepleting effects might prevent the viability and effective anticancer properties of adoptive immune cells if administered at the same time. To characterize the vulnerability of unarmed eNK and Pin-EGFR-armed eNK cells to TMZ, their viability was first evaluated over time in the presence of increasing TMZ concentrations. As depicted in Figure 6A, exposure to pharmacological doses of TMZ had no significant impact on eNK-cell viability even after 72 h. The effect of TMZ on cell functionality was then assessed by analyzing the impact of increasing doses on the cytotoxicity of unarmed eNK cells and Pin-EGFR-armed eNK cells (Figure 6B). While TMZ itself induced an expected dose-dependent decrease in the viability of U-87 MG cells, it had no impact on the efficacy of armed cells, even at the highest concentration. Furthermore, when TMZ and Pin-EGFR-armed eNK cells were combined, an additive effect on target cell viability seemed to emerge.

Consequently, the possible simultaneous therapeutic use of Pin-EGFR-armed cells and TMZ is not excluded. Further analyses will be needed to determine whether this combination could provide an additive, or even synergistic, effect.

## 4. Discussion

In this study, we provide experimental evidence indicating that the use of a modified anti-EGFR antibody pre-complexed with donor-sourced NK cells represents a major opportunity in glioblastoma settings. As a proof of concept, in this in vitro study, we show the preliminary development of a novel allogeneic human cell and antibody-based drug candidate, with significant targeted potency against GBM cells expressing EGFR.

In contrast to the main vision of CAR (chimeric antigen receptor) genetically modified NK cells with improved functional activity, we hypothesized that NK cells can be directed against specific targets by using their low-affinity CD16 receptor without any genetic modification. The introduction of limited mutations in the Fc portion of the anti-EGFR (cetuximab) monoclonal antibody, creating the so-called Pin-EGFR, was shown to improve recognition by CD16 and, hence, allow for the long-term stabilization of the mAb on the NK plasma membrane. Consequently, we demonstrated that Pin-EGFR mAbs can be noncovalently ‘armed’ onto NK cells, which is not possible with unmodified mAbs. In contrast to CAR-NK cells, our strategy offers multiple advantages, including the absence of artificial receptors induced by genetic modifications requiring complex manufacturing [42,43]. It also offers greater flexibility, since the targeting moiety can be easily interchanged. Other technologies to pre-arm allogeneic NK cells have been suggested to overcome CAR-NK-cell drawbacks: pre-complexing NK cells with multi-specific constructs targeting at least one NK receptor and one tumor antigen or performing chemical antibody conjugation on cells [44,45,46,47,48]. Although effective, these technological platforms still require the functionalization of the NK-cell surface or the development and manufacture of complex molecules and are not based on a fully natural mechanism of action. Furthermore, very few of these pre-complexing technologies focus on combining NK cell-based therapies with EGFR-targeted molecules, and none have been investigated in brain tumors.

We observed that most of the loss of arming occurs within 24 h following preparation. Since the CD16 expression is stable, this loss of arming may not be due to CD16 cleavage or internalization. This finding further suggests that the noncovalent binding remains labile, opening a new way of optimizing CD16/Pin-mAb interactions by modifying the mAb structure [49,50,51]. Nevertheless, it is important to highlight that, in a clinical context, this loss of antibody binding is not problematic and may even be an advantage. First, as we have shown, ADCC induced by armed cells occurs very rapidly, in just a few hours, suggesting that the arming retention time should be sufficient and consistent with administration to patients. Second, any intact mAbs that detach from the NK cells will still be able to bind to their targets (Fc receptors and EGFR), enabling a second theoretical therapeutic effect by re-engaging eNK cells and/or engaging the patient’s own immune cells. In any case, considering the short lifespan of NK cells, and like most therapies developed using unmodified NK cells, repeated doses will be necessary to maximize patients’ chances of remission [52].

We showed that armed NK cells are not activated for effector actions until the armed antibody encounters its target, indicating that cell activation occurs only when the armed antibody forms a normal immune synapse, preventing the loss of potency prior to tumor contact.

With cytotoxic activity against GBM cells occurring rapidly following therapeutic cell contact and the release of IFN-γ and TNF-α cytokines, the ADCC-like property of pre-armed eNK cells resembles the natural conventional ADCC property of NK cells in the presence of mAb-opsonized target cells [53,54,55]. Consequently, arming immune cells induces an ADCC-like process that reconsiders the paradigm according to which ADCC-exhibiting mAbs first encounter their antigen on target cells before engaging the immune system. Furthermore, the very rapid induction of target cell death suggests that extended therapeutic cell survival is not needed for the high potency of Pin-EGFR-armed eNK cells. We further demonstrated that such armed cells can be effective in vitro at specifically and substantially destroying all cancer cells expressing the target ligand from a single treatment using conventional GBM cell lines and patient-derived GBM tumor samples at ratio of therapeutic to target cells that will be feasible for therapy.

Moreover, we showed that arming NK cells can overcome variability in donor characteristics. This is consistent with evidence that negative signals transmitted by tumor cells can attenuate NK-cell responses and that of all the activating receptors present on NK cells, mAb-derived CD16 is the most likely to overcome inhibitory signals [56,57]. Consequently, the arming of NK cells prior to their administration to the patient can be considered a convergent manufacturing method, not only allowing any donor to be qualified without specific selection but also potentially providing greater homogeneity in responses to treatment.

NK- and T-cell therapies have demonstrated limited efficacy in solid tumors, including GBM. One of the most important reasons is the immunosuppressive tumor microenvironment (TME), which promotes tumor growth and suppresses the immune cells used to eliminate tumor cells [58]. Human transforming growth factor-β (TGF-β) plays a crucial role in forming this suppressive GBM TME and driving the suppression of the anti-GBM response [59]. To mitigate TGF-β-mediated suppressive activity, pharmacological TGF-β antagonists can be combined with cell therapy, or the cell therapy itself can be engineered to become resistant to TGF-β action [37,60,61]. However, unexpectedly, Pin-EGFR-armed eNK cells retain their full functional activity in an in vitro environment containing TGF-β, reproducing/mimicking the immunosuppressive tumor microenvironment. It should be pointed out that our study is the first to assess the effect of TGF-β in the context of UCB-derived NK cells expanded using our own method. The source of NK cells, the donors and the specific process used to expand and activate the cells may be partly responsible of their observed low sensitivity to TGF-β compared with other published data [62]. One potential reason could be a phenotypic difference, such as the level of expression of the TGF-β receptor [63]. Another aspect concerns the different experimental conditions in past studies, with many cases where NK cells were exposed to TGF-β for several days to visualize its impact. To conduct a comprehensive analysis, different NK sources and donors should be assessed for their TGF receptor expression and their functionality (cytotoxicity and cytokine secretion) in the presence of TGF-β. Although Pin-EGFR-armed eNK cells will act in a short period of time once engaged with their target cells, studying the effect of longer incubation times in the presence of TGF-β could also provide relevant mechanistic information.

Finally, we investigated the impact of temozolomide on eNK cells, the standard of care for GBM patients known to induce strong lymphodepletion in addition to toxicity toward cancer cells. As previously shown in local and peripheral NK cells that demonstrate resistance to chemotherapy [64], we also confirmed that neither NK-cell viability nor function is compromised in the presence of pharmacological doses of temozolomide, guaranteeing their functionality in specific clinical cases where the drug would be concomitantly used.

Despite decades of unrelenting efforts by the research and medical communities to combat this disease, including advances in neurosurgery, chemotherapy and radiotherapy, GBM still remains one of the most treatment-resistant and lethal CNS malignancies, and the tumor inevitably recurs. Indeed, few new therapies have shown efficacy for mitigating glioblastoma since the introduction of temozolomide as part of the Stupp protocol in 2005 [65]. The resection cavity is an important site to prevent early tumor recurrence and, the isolation of the brain by the BBB creates a unique opportunity to deliver aggressive treatment locally with a limited risk of systemic toxicity. Thus, many new therapies focus on combatting these local recurrences by implementing treatments directly in or near the tumor bed. Many phase 1 and 2 trials experimenting with various forms of local therapy have been—and are being—conducted in glioblastoma, with many showing great potential for improving progression-free survival (PFS) [66,67]. Aside from direct anti-tumor effects, local therapy might induce and enhance the local immune response, improving the efficacy of therapies that have shown limited benefits when administered systemically (such as antibodies and even temozolomide, for which a clinical trial of intra-arterial delivery recently completed recruitment, NCT01180816) [68]. This is particularly true in the case of cellular therapies, where peripherally infused CAR T cells targeting EGFRvIII failed in a pilot trial (NCT01454596) [12,13]. Cell therapies will be able to demonstrate their full effect in local therapy thanks to their capability to act locally but also migrate throughout the brain and attack distant foci of tumor cells. Preclinical studies have established that, based on cell dose, locoregional delivery is more effective than systemic delivery for this regionally restricted central nervous system tumor [69,70,71]. Consequently, the locoregional delivery of CAR T cells, in which the therapeutic cells are delivered via a reservoir (Ommaya)/catheter device into either the tumor bed, tumor resection cavity or cerebrospinal fluid (CSF), has already been applied in clinical settings [72]. Our study supports an approach to pre-complexing ex vivo normal expanded donor immune cells and Fc-engineered human monoclonal antibody molecules, resulting in the self-assembly of potent cytotoxic entities, with the local infusion of this armed product into the patient. Our results highlight several achievable characteristics of Pin-EGFR-armed immune cells that would be compatible with the use of the product within a coherent local treatment protocol for GBM patients. Treatment at the tumor bed to supplement surgical debulking immediately after surgery and in the post-surgical recovery period, where chemotherapy and radiation treatments are impractical, must enhance current GBM therapy principally by acting to further deplete tumor cell numbers/residual tumor cells with a low risk of side effects on patients. In addition, this approach would make it possible to extend the strategy of eliminating the supramarginal zone to a larger number of patients, or even to the majority of patients, since this precise and complex surgery remains limited to a few patients because of the risk of neurological loss of function due to over-invasive resection. Taken together, our findings reveal the potential of a new therapeutic strategy aiming at combining EGFR-targeted therapies and immune cells at the tumor bed to overcome resistance and recurrence by effectively removing residual tumor tissue before the reorganization and re-establishment of tumor proliferation.

One of the major limitations of the broader applicability of allogeneic adoptive cell therapies, and particularly their persistence, is immune rejection, known as the host-versus-graft (HvG) reaction. MHC (major histocompatibility complex) compatibility plays a critical role in immune response. The loss of self-recognition by MHC class I molecules or differences in MHC class II molecules between adoptively transferred cells and host immune cells may be responsible for the depletion or clearance of transferred therapeutic cells. Several strategies have been actively investigated to evade the allo-rejection of cell therapy products, including pharmacologic immune suppression, HLA (human leukocyte antigen) matching and gene editing to deplete MHC class I and II molecules [73,74,75]. Unfortunately, none of these strategies is currently unanimously accepted for the use of allogeneic NK cells to treat solid tumors. Our approach could bypass this problem of persistence given the rapid therapeutic effect of the Pin-EGFR-armed eNK cells and the local application of repeated-frequency injections. In the future, in vivo studies in a clinical setting will thus be conducted to elucidate whether the promising in vitro observations of Pin-EGFR-armed eNK cells are reproducible in patients for eliminating post-surgical residual tumor cells. Armed NK cells may have the potential to become an important part of the repertoire of therapeutic interventions for GBM and could result in both improved survival and an enhanced quality of life in patients stricken with this serious condition. Furthermore, the modular nature of the product concept means that second-generation developments, including multitargeting and target switching to address tumor evasion and escape, are straightforward to implement, enabling future clinicians to potentially stay one step ahead of tumors’ survival tactics. We have started to investigate this approach with Pin-HER2 targeting HER2, another member of the ErbB family, the high expression of which has been associated with the development and progression of GBM [76]. Promising preliminary data are in favor of treating GBM cells with the sequential use of Pin-EGFR-armed, Pin-HER2-armed or Pin-EGFR/Pin-HER2-armed cells (yet unpublished results).

## 5. Conclusions

In conclusion, we have developed a novel approach to GBM immunotherapy using allogeneic umbilical cord blood-derived and expanded NK cells complexed with modified monoclonal antibodies bound to the intrinsic Fc receptor. This study provides evidence of the superior efficacy of pre-armed eNK cells with Fc-engineered monoclonal antibodies in inducing ADCC when compared with the efficacy of unarmed eNK cells. The magnitude and specificity of ADCC induced by the pin-EGFR-armed eNK cells remain unaffected in an immunosuppressive environment mimicking the TME of GBM. These results suggest that pre-complexed immune cells may be amenable to further development as allogeneic off-the-shelf cell products.

## 6. Patents

J.P. is a co-inventor of WO2022023581 describing the approach of arming CD16-positive immune cells with Fc-engineered constructs.

## Data Availability

The original contributions presented in this study are included in the article. Further inquiries can be directed to the corresponding author.
